# Targeted Extracellular Vesicles Delivered Verrucarin A to Treat Glioblastoma

**DOI:** 10.3390/biomedicines10010130

**Published:** 2022-01-07

**Authors:** Kai Chen, Yingnan Si, Jia-Shiung Guan, Zhuoxin Zhou, Seulhee Kim, Taehyun Kim, Liang Shan, Christopher D. Willey, Lufang Zhou, Xiaoguang Liu

**Affiliations:** 1 Department of Biomedical Engineering, University of Alabama at Birmingham (UAB), 1825 University Blvd, Birmingham, AL 35294, USA; kaisdzb@uab.edu (K.C.); yingnan@uab.edu (Y.S.); zhouzhx@uab.edu (Z.Z.); 2 Department of Medicine, University of Alabama at Birmingham (UAB), 703 19th Street South, Birmingham, AL 35294, USA; guan0926@uab.edu (J.-S.G.); seulheekim@uabmc.edu (S.K.); kimth@uab.edu (T.K.); 3 School of Nursing, University of Alabama at Birmingham (UAB), 1701 University Blvd, Birmingham, AL 35294, USA; lshan@uab.edu; 4 Department of Radiation Oncology, University of Alabama at Birmingham (UAB), 1700 6th Avenue South, Birmingham, AL 35294, USA; cwilley@uabmc.edu

**Keywords:** glioblastoma, targeted delivery, monoclonal antibody-directed extracellular vesicle, natural compound verrucarin A

## Abstract

Glioblastomas, accounting for approximately 50% of gliomas, comprise the most aggressive, highly heterogeneous, and malignant brain tumors. The objective of this study was to develop and evaluate a new targeted therapy, i.e., highly potent natural compound verrucarin A (Ver-A), delivered with monoclonal antibody-directed extracellular vesicle (mAb-EV). First, the high surface expression of epidermal growth factor receptor (EGFR) in glioblastoma patient tissue and cell lines was confirmed using immunohistochemistry staining, flow cytometry, and Western blotting. mAb-EV-Ver-A was constructed by packing Ver-A and tagging anti-EGFR mAb to EV generated from HEK293F culture. Confocal microscopy and the In Vivo Imaging System demonstrated that mAb-EV could penetrate the blood–brain barrier, target intracranial glioblastoma xenografts, and deliver drug intracellularly. The in vitro cytotoxicity study showed IC_50_ values of 2–12 nM of Ver-A. The hematoxylin and eosin staining of major organs in the tolerated dose study indicated minimal systemic toxicity of mAb-EV-Ver-A. Finally, the in vivo anti-tumor efficacy study in intracranial xenograft models demonstrated that EGFR mAb-EV-Ver-A effectively inhibited glioblastoma growth, but the combination with VEGF mAb did not improve the therapeutic efficacy. This study suggested that mAb-EV is an effective drug delivery vehicle and natural Ver-A has great potential to treat glioblastoma.

## 1. Introduction

Gliomas are the most prevalent primary intracranial cancer, including the highly malignant, aggressive, heterogeneous, and angiogenetic glioblastomas (GBM, WHO grade IV), which account for the majority of gliomas [1]. Surgery followed by the combination of involved-field radiation therapy and the DNA alkylating agent chemotherapy temozolomide (TMZ) is the current standard treatment strategy for newly diagnosed GBM in clinics [2,3]. Due to the stem-like cells, blood–brain barrier (BBB), or hypoxia, GBMs are usually resistant to conventional therapies with a high recurrence rate. The United States Food and Drug Administration has approved bevacizumab, an anti-vascular endothelial growth factor (VEGF) monoclonal antibody (mAb), to treat recurrent GBM. Despite the progress, the current standard care only provides a median survival of 14.6 months for GBM patients [4].

As reviewed before, multiple therapies have been developed to target the core signaling pathways that play important roles in GBM development [5]. For example, epidermal growth factor receptor (EGFR) inhibitors (TK1, neratinib, cetuximab, rindopepimut) [5,6,7,8], anaplastic lymphoma kinase inhibitors (imatinib and crizotinib) [9,10,11], vascular endothelial growth factor (VEGF) inhibitors (bevacizumab, vatalanib, tivozanib, cediranib) [12,13,14], platelet-derived growth factor receptor (PDGFR) inhibitors (Sunitinib and Nintedanib) [15,16], phosphoinositide 3-kinase (PI3K)/protein kinase B (AKT)/mammalian target of rapamycin (mTOR) inhibitors (Sonolisib, Temsirolimus, Sirolimus, Everolimus, Vextalisib) [17,18], p53 restoration gene therapy (miRNA) [19], and retinoblastoma tumor suppressor (RB) pathway CDK4/6 inhibitors (Palboclib) [20] have been developed and/or evaluated in clinical trials. Many of these therapies have failed in clinical trials due to the challenges of poor anti-tumor efficacy, development of drug resistance, and BBB. Therefore, new therapeutics and an efficient drug delivery vehicle are highly desired for GBM treatment.

The natural compound verrucarin A (Ver-A, a type D macrocyclic trichothecene), which is isolated from the metabolites secreted by *Myrothecium verrucaria* [21], has been reported as a highly potent therapy to treat cancers. For instance, previous studies demonstrated that Ver-A had strong antiproliferative and proapoptotic effect on renal [22], hepatocellular [23], leukemia [24,25], and breast [26] carcinoma by blocking cell cycle progression via inhibiting cell cycle regulatory proteins cyclin D1/E, cyclin-dependent kinases inhibitor WAF1/21, or protein kinase B (AKT)/nuclear factor kappa B (NF-κB)/mammalian target of rapamycin (mTOR) prosurvival signaling, or by inducing apoptosis [27]. Moreover, our previous study showed that Ver-A can effectively inhibit neuroendocrine tumor growth [28]. The anti-GBM efficacy of Ver-A has not been investigated in GBM treatment so far.

As reviewed by Samec et al., targeted nanoparticles have been developed to deliver therapies to treat GBM [29], including liposomes carrying doxorubicin or irinotecan [30], polymeric nanoparticles delivering paclitaxel or miRNA mimics [31,32], solid lipid nanoparticles loaded with temozolomide and vincristine [33], polymeric micelles delivering doxorubicin and curcumin [34], and dendrimers bearing doxorubicin and siRNA [35]. These delivery vehicles have been limited in clinic application due to the poor stability, limited loading capacity, toxicity or reduced efficacy because of BBB. Recent studies showed that extracellular vesicle (EV), a natural endogenous nanoparticle, has great potential for targeted delivery of highly potent drugs with the advantages of immune tolerance, circulation stability, capability to cross BBB, and tumor targeting via surface-tagged GBM-targeted reagents [36,37]. Our previous studies have established the platform of EV biomanufacturing and surface tagging [38] and demonstrated its capability of targeted delivery of combined therapies [28]. Furthermore, we have demonstrated and reported that the monoclonal antibody (mAb) is effective to target tumor in vivo [39,40,41,42] or direct drug delivery vehicles such as liposomes and EVs to target tumors [30,40]. Therefore, mAb-EV was constructed to deliver payload to GBM.

The literature has reported multiple growth factors or cytokines surface receptors overexpressed in GBM [43,44], including EGFR, PDGFR, VEGFR, transforming growth factor-βR, hepatocyte growth factor/scatter factor receptor, G protein-coupled receptor, interleukin-4, 13, or Urokinase-type plasminogen activator receptor. As an important signal in the receptor tyrosine kinase (RTK) pathway, EGFR or variant III (EGFRvIII) is overexpressed in over 40% of GBM patients [45] and is an attractive target for the development of targeted therapies, such as geftinib, neratinib, and CAR-T cells [5]. The chimeric anti-EGFR mAb (cetuximab) showed strong binding to EGFR and EGFRvIII, which was tagged on the surface of EV to target GBM in this study.

This study aimed to develop and evaluate a new targeted therapy, i.e., mAb-EV-delivered Ver-A, to treat the malignant EGFR-positive GBMs. The high surface expression of EGFR in GBM patient tissues and cell lines was confirmed. The mAb-EV-Ver-A was constructed and characterized. The GBM targeting, drug delivery, toxicity, and anti-tumor efficacy of the developed therapy were evaluated using cell lines and intracranial xenograft mouse models. Our study showed that the anti-EGFR mAb-EV-Ver-A can effectively target GBM and inhibit the tumor growth with minimal toxicity.

## 2. Materials and Methods

### 2.1. Cell Lines, Seed Cultures, and Media

The human GBM cell lines, including malignant U251 (MilliporeSigma, Manassas, VA, USA), U87 (ATCC, Manassas, VA, USA), and U87-FLuc (ATCC), were used to evaluate the developed targeted therapy. The U251 cells were maintained in EMEM (Gibco, Grand Island, NY, USA) supplemented with 2 mM L-glutamine, 1% non-essential amino acids (NEAA), 1 mM sodium pyruvate, 10% fetal bovine serum (FBS), and 1% penicillin/streptomycin (P/S) in T25 or T75 flasks (Fisher Scientific, Waltham, MA, USA). The U87 and U87-FLuc cells were maintained in EMEM supplemented with 10% FBS and 8 µg/mL Blasticidin. The normal human astrocyte cell line NHA (Lonza, Greenwood, SC, USA) and the negative control of GBM were maintained in DMEM (Gibco) containing 10% FBS. All cell cultures were maintained in CO_2_ incubator (Caron, Marietta, OH). The cell growth was monitored by analyzing viable cell density (VCD) and viability.

### 2.2. Intracranial Xenograft Model

The five-week-old NSG (NOD.Cg-Prkdc<scid> Il2rg<tm1Wjl>/SzJ) mice were purchased from Jackson Laboratory (Bar Harbor, ME, USA) to generate a GBM intracranial xenograft model. A total of 5 × 10^5^ of U87-FLuc cells were stereotactically injected into mice using the Stereotaxic Instrument (Fisher Scientific) following our reported protocol [39].

### 2.3. Construction of mAb-EV-Drug

As detailed in our previously published platform [38], EV was produced by HEK293 cells in a FreeStyle^TM^ 293 expression medium (Gibco). Briefly, the 250 mL of basal medium supplemented with 6 g/L glucose, 6 mM L-glutamine, and 3.5 g/L Cell Boost 6 was inoculated with HEK293 cells with a seeding density of 0.3 × 10^6^ cells/mL in 1 L shaker flasks. The EV production culture was incubated at 37 °C and an agitation of 80 rpm. The spent medium was collected when cell viability dropped <80%. The harvested EV was purified with the size exclusion column of Vivaspin 300 kDa MWCO or the fast flow affinity purification column packed with NHS-activated Sepharose (Cyvita, Marlborough, MA, USA), which was coupled with an anti-CD63 antibody (BioLegend, San Diego, CA, USA). The EV purity isolated with size exclusion purification is enough to deliver drugs in vivo, as validated in our previous study [38]. The generated EV was titrated with NanoSight (Malvern Panalytical, Malvern, UK) and characterized with Western blotting. Then, the mAb-EV was constructed by tagging anti-EGFR mAb to EV via DSPE-PEG-NHS linker and modified with mPEG-DSPE to improve its circulation stability following our established procedure [38]. The literature also reported that the PEG tagged on the surface of EV could provide stealth property to “evade” the immune response [38], reduce the clearance rate, and extend the circulatory half-life [46] of the engineered EV. The untagged mAb, linker, and stabilizer were removed using a Vivaspin 300 kDa MWCO column. Finally, the mAb-Exo-drug was generated by incubating a 10 × 10^10^ particle (ptc) of EV with 0.101 mg (200 nanomole) of Ver-A in 8 mL of PBS overnight at room temperature, and the unpacked free drug was removed with the Vivaspin 100 kDa column. The mAb-EV (1 × 10^12^ ptc) was labeled with Liss Rhod or Cy7 fluorescent dye (16.7 nmol) via mPEG-1, 2-Distearoyl-sn-glycero-3-phosphoethanolamine (DSPE, 2 µmol) to monitor the in vitro drug uptake or in vivo drug biodistribution, respectively.

### 2.4. In Vitro Anti-GBM Cytotoxicity

To test in vitro anti-GBM cytotoxicity [47], the U251 or U87 cells were seeded in 96-well plates with a density of 5 × 10^4^ cells/mL in 200 µL of growth medium and incubated for 24 h. The 5-day treatment was performed by adding free TMZ (0, 10, 25, 50, 75, 100, 150, or 200 µM), free Ver-A (0, 5, 10, 15, 20, 30, 40, or 50 nM), or combined TMZ (0–50 µM) and EV-Ver-A (0–10 nM). The treated cells were washed using PBS, and 100 µL fresh culture medium was added to the wells after wash. Then TACS 2,5-diphenyl-2H-tetrazolium bromide (MTT) Cell Proliferation Assay (R&D Systems, Minneapolis, MN, USA) was performed to test the relative cell viability by adding 10 μL of MTT reagent to each well to develop a purple color, adding 100 μL of detergent reagent, and reading OD values at 570 nm, which is proportional to the viable cell number. The IC_50_ values were calculated using ED50V10 Excel add-in.

### 2.5. Patient Tissue Array (TMA) and Immunohistochemistry (IHC) Staining

The brain GBM tissue arrays, including 35 cases and 70 cores, were purchased from US Biomax (Derwood, MD, USA). IHC staining was performed to identify the surface receptor in patient tissues as we described before [40,48]. The TMA slides were stained with rabbit anti-human EGFR mAb (Abcam, Waltham, MA, USA) and counterstained with hematoxylin.

### 2.6. Western Blotting

Western blotting analysis was performed to analyze EGFR expression and the proliferation markers, such as Cyclin D1, p21, and p27, and post mAb-EV-Ver-A treatment in U87 and U251 cell lines was performed following previously established protocols [28,38].

### 2.7. Flow Cytometry Analysis

The GBM surface binding rate of anti-human EGFR mAb in U87 and U251 cells was evaluated and quantitated using a BD LSRII flow cytometer (BD Biosciences, San Jose, CA, USA) following our published methods [41]. Briefly, the mAb was labeled with Alexa Fluor™ 647 labeling kit. The 1 × 10^6^ of GBM cells were collected, washed, and re-suspended in 100 µL of PBS containing 1% FBS, and incubated/stained with 1 µg of AF647-EGFR mAb at room temperature for 30 min. The stained cells were washed three times with PBS and analyzed with a flow cytometer. The absolute cutline of negative staining, i.e., glioblastoma cells without antibody staining, was used in gating.

### 2.8. Transmission Electron Microscope (TEM)

The TEM image was taken to confirm the isolated EVs. First, the EV sample was solved in 10 mM Tris buffer and concentrated with 300 kDa column. Second, the formvar-carbon-coated grid was discharged through K100X Glow Discharge with parameters of 50 mA and 20 s. Third, the EV sample was applied to grid for 1 min and negatively stained with 1% filtered uranyl acetate. Fourth, the sample on the grids was imaged with a Tecnai T12 transmission electron microscope (FEI, Hillsboro, OR, USA) equipped with AMT CCD camera.

### 2.9. Confocal Imaging

The in vitro uptake of mAb-EV by GBM cell lines was confirmed with two-color confocal microscopy imaging [28]. Briefly, the mAb-EV was stained with Liss Rhod fluorescent dye (red) with mAb-EV:Liss Rhod:mPEG-DSPE molar ratio of 1:10,000:300,000 at room temperature with overnight horizontal shaking in the dark. The free dye was removed by a 100 kDa MWCO concentrator. The U251 cells were seeded in a chambered glass coverslip with viable cell density of 1 × 10^5^ cells/mL, and the cytoplasm and nucleus of cells were infected and stained with BacMam GFP Transduction Control (Green) at MOI of 50 for overnight. Then, mAb-EV-Liss Rhod was mixed and incubated with GBM cells overnight. After washing with the fresh cell growth medium, the live-cell images were collected using Nikon A1R-HD25 confocal microscope with a high-speed resonance scanner (Nikon USA, Melville, NY, USA).

### 2.10. In Vivo Imaging System (IVIS) Imaging

The tumor growth of U87-FLuc cell line-derived intracranial xenografted NSG mouse model was monitored by measuring bioluminescent signal (FLuc) with IVIS Lumina Series III (PerkinElmer, Waltham, MA, USA) every three to four days post tumor cell implantation. To confirm the in vivo GBM targeting and the capability to penetrate the BBB of the mAb-EV-drug, 3 × 10^11^ particles (ptc) of Cy7-labeled mAb-EV or 50 µg of Cy5.5-labeled mAb was intravenously (i.v.) injected into mice via the tail vein. Then, the xenograft mice were imaged under IVIS to capture the tumor bioluminescence (FLuc) at a wavelength of 550 nm and the targeted delivered fluorescence (Cy7) at a wavelength of 750 nm of xenograft binding at 24 h post injection. The in vivo biodistribution and GBM-targeting of mAb or mAb-EV was analyzed by detecting the co-localization of FLuc and Cy7 signals. Furthermore, the important organs, such as the brain, heart, lung, kidney, and spleen, were also extracted to collect ex vivo images to check the possible off-target binding.

### 2.11. Tolerated Dosage (TD) Study

To investigate the tolerated dosages of targeting delivered Ver-A and its potential toxicity, six doses of mAb-EV-Ver-A (i.e., 0, 1, 2, 3, 4, and 5 mg/kg) were i.v. injected into the non-tumor bearing BALB/cJ mice (Jackson Laboratory) via the tail vein (*n* = 2). The body weight of mice was monitored every 2 days for a total of 14 days. All mice showed no overt changes in general health and body weights (>20%). At the end of the study, the mice were sacrificed to collect the major organs, including the brain, heart, lung, liver, kidney, and spleen, for hematoxylin and eosin (H&E) staining to analyze the potential toxicity of mAb-EV-Ver-A.

### 2.12. In Vivo Anti-GBM Efficacy Study

The U87-FLuc intracranially xenografted NSG mice were randomized into 4 groups (*n* = 5) when the FLuc bioluminescence intensity was higher than 20,000 in IVIS imaging on day 7 post transplantation. To mimic clinical treatment, all xenograft mice were treated with 1 mg/kg of free TMZ via i.p. injected daily on days 7–9. Then the mice were treated with mAb-EV (negative control), 1.0 mg/kg of mAb-EV-Ver-A, 3.0 mg/kg of mAb-EV-Ver-A, and 3.0 mg/kg of mAb-EV-Ver-A in combination with 5 mg/kg of anti-VEGF mAb via tail vein injection on a Q3/7Dx6 schedule (3/7-day interval for 6 injections). The body weight and tumor volume were monitored every 3–4 days. The mice were sacrificed when we observed slow locomotion and obvious body weight drop in the control group.

### 2.13. Hematoxylin and Eosin (H&E) Staining

The sections of major organs harvested from the in vivo treatment with mAb-EV-Ver-A were stained with H&E. The detailed staining procedure has been reported in our previous publications [40,48].

### 2.14. Statistical Analysis

The experimental data were presented as mean ± standard error of the mean (SEM). Two group comparisons were performed using unpaired Student’s *t* test to determine the probability of significance. Comparison was performed using a one-way ANOVA followed by post-hoc (Dunnett’s) analysis. The sample size in animal study was determined following our previous therapy study [41]. Statistical significance with ** *p* value of <0.005 was considered for all tests.

## 3. Results

### 3.1. EGFR Surface Expression in GBM

**Figure 1:**
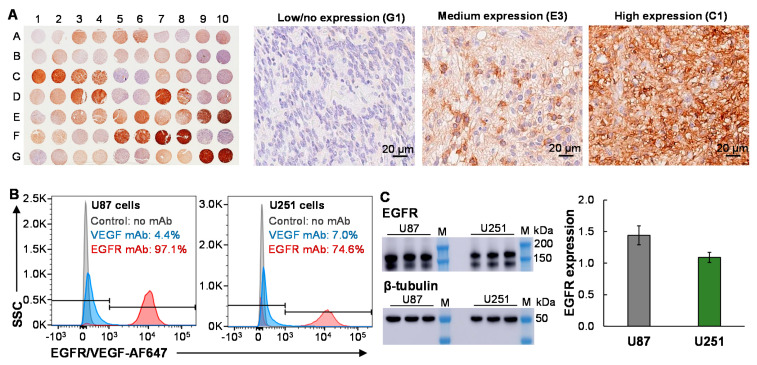
EGFR expression in GBM. (**A**) IHC staining of patient tissue microarray (TMA) to analyze EGFR surface expression in GBM (35 cases, 70 cores). Scale bar equals 20 µm. (**B**) Evaluation of surface binding rate of VEGF mAb-AF647 (blue) or EGFR mAb-AF647 (red) in GBM U87 and U251 cells by flow cytometry analysis. One million cells were stained with 1 μg of mAb-AF647 at room temperature for 30 min. (**C**) Western blotting analysis of EGFR in two GBM cell lines. 1: U87; 2: U251; and 3: marker.

To assess the surface receptor expression of EGFR, GBM tissue microarray slides (35 GBM cases and 70 cores) were performed with IHC staining (Figure 1A). The relative expression level of receptor was analyzed with ImageJ and quantitated using the score of ratios of DAB intensity: nuclei intensity. The IHC staining showed that 54% (38 of 70) of patient tissue samples had high (score of >10) or medium (score of 5–10) expression with cell membrane localization, 34% (24 of 70) of samples had low expression (score of 1–5), and 8% (8 of 70) of samples had no or minimal expression (score < 1). The representative images of IHC staining with high (C1), medium (E3), and low/no (G1) expression are presented in Figure 1A. The IHC staining of the adjacent normal brain tissues has low or minimal expression, indicating that EGFR is a good target of GBM. These data indicated that the EGFR-targeted EV-drug could cover >53% patients with malignant GBM. For the EGFR-GBM patients, we can consider targeting an alternative receptor, such as CXCR4 or CD276, which was reported to overexpress in GBM patient tissues [49,50].

Furthermore, the surface expression of EGFR in malignant GBM cell lines was evaluated by staining U87 and U251 cells with an antibody at room temperature. As presented in Figure 1B, flow cytometry analysis showed that the U87 and U251 cells had high EGFR expression with a binding rate of 97.1% and 74.6%, respectively (Figure 1B). The VEGF expression in these cell lines was low with a binding rate of 4.3%–7.0%. Finally, the EGFR expression was confirmed using Western blotting (Figure 1C). Although the predicted size of human EGFR was 134 kDa, two bands were detected by Abcam primary anti-human EGFR antibody with observed sizes of ~130 kDa and ~170 kDa in U87 and U251 cells, respectively. The possible root cause of changed molecular weight was high post-translational modifications or alternative splice variants of EGFR. The calculated relative expression (i.e., receptor intensity/β-tubulin intensity) of U87 and U251 was 1.43 and 1.05, respectively.

### 3.2. Construction of Targeted mAb-EV-Drug

**Figure 2:**
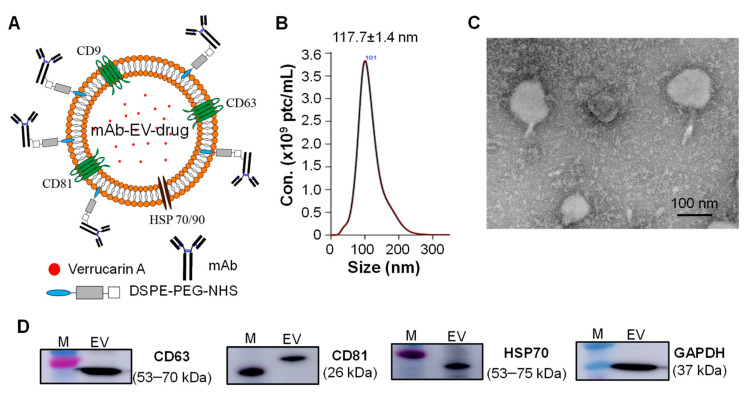
Characterization of mAb-EV-drug. (**A**) Construction and structure of mAb-EV-Ver-A: mAb was surface tagged on EV via DSPE-PEG-NHS and payload Ver-A was packed in EV. (**B**) Size distribution by NanoSight assay. (**C**) TEM image of mAb-EV-Ver-A. Scale bar equals 100 nm. (**D**) Western blotting analysis of EV biomarkers (CD63, CD81, HSP70, and GAPDH).

The EGFR mAb-EV-Ver-A (Figure 2A) was constructed following our previously established platforms of EV production, surface labeling, and drug packing [28,38]. The size distribution of the targeted vehicle (mAb-EV-Ver-A) was analyzed using NanoSight assay, demonstrating a homogenous distribution with an average diameter of 117.7 ± 1.4 nm (Figure 2B). The vehicle was further confirmed with transmission electron microscopy (TEM) image (Figure 2C). The purified EV was confirmed with Western blotting by analyzing the biomarkers of surface tetraspanins (CD63 and CD81), heat shock protein 70 (HSP70), and glyceraldehyde 3-phosphate dehydrogenase (GAPDH), as presented in Figure 2D. The constructed mAb-EV-drug was further evaluated by testing the GBM targeting, toxicity, and anti-tumor cytotoxicity or efficacy in the following studies.

### 3.3. GBM Targeting by mAb-EV

**Figure 3:**
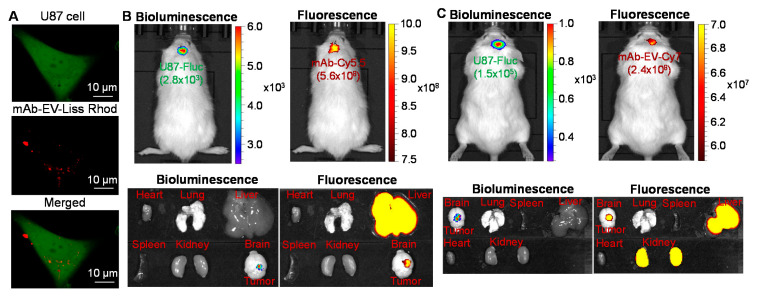
Evaluation of GBM targeting of mAb-EV. (**A**) Live-cell CLSM imaging of mAb-EV-Liss Rhod internalization in U87 cells. Two-color CLSM: whole cell labeled with GFP (displayed as green) and mAb-EV labeled with Liss Rhod (displayed as red). Scale bar equals 10 µm. (**B**) In vivo live animal and ex vivo IVIS imaging of tumor and important organs to analyze tumor targeting and biodistribution of mAb-Cy5.5 at 24 h post i.v. injection in U-87-FLuc intracranial xenograft mouse model (*n* = 3). (**C**) IVIS imaging of the live animal and tumor and organs to analyze tumor targeting and biodistribution of mAb-EV-Cy7 at 24 h post i.v. injection (*n* = 3).

The confocal laser scanning microscopy (CLSM) imaging was used to test the in vitro targeted delivery capability of EGFR mAb-EV. As shown in Figure 3A, the mAb-EV-Liss Rhod can internalize into the cytoplasm of U87 cells and deliver the packed fluorescent dye (displayed as red color) or drugs intracellularly. The in vivo GBM specificity and biodistribution of EGFR mAb-Cy5.5 and mAb-EV-Cy7 were evaluated in U87-FLuc intracranially xenografted mouse model via i.v. injection. As described in Figure 3B, the live-animal IVIS imaging at 24 h demonstrated that the bioluminescent FLuc (tumor) and fluorescent Cy5.5 (mAb) overlapped in the brain, which suggested that the EGFR mAb penetrated the BBB and accumulated in the GBM xenograft. The ex vivo imaging of the important organs, such as the heart, lung, liver, spleen, kidney, and brain, confirmed the tumor targeting of the mAb, but also showed distribution of Cy7 in the liver. Furthermore, the capability of GBM targeting of mAb-EV-Cy7 was assessed and confirmed in the same animal model using IVIS imaging (Figure 3C). The ex vivo images also indicated the distribution of mAb-EV-Cy7 in the liver and kidney, probably due to the metabolism of Cy7 dye, which needs further investigation. These data showed that both mAb and mAb-EV can penetrate the BBB, target the GBM xenograft, and deliver the packed cargos.

### 3.4. In Vitro Anti-Cancer Cytotoxicity

**Figure 4:**
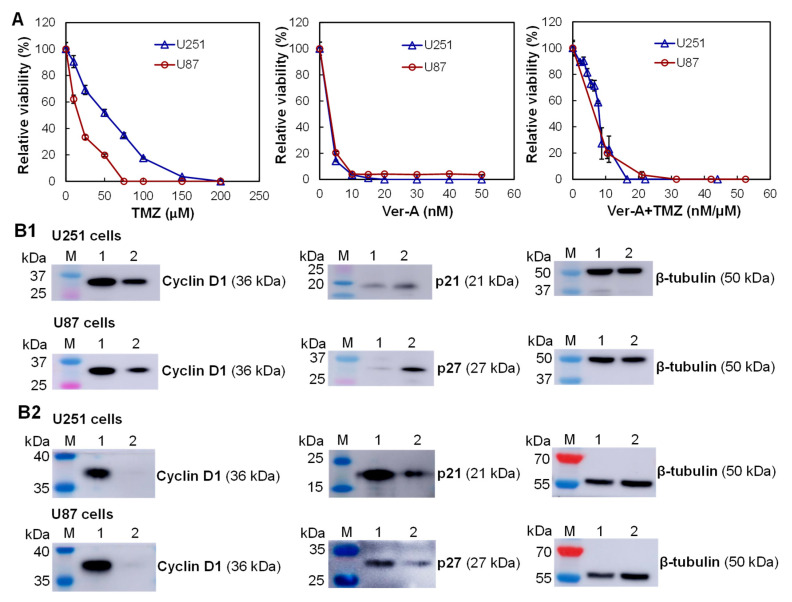
In vitro anti-GBM cytotoxicity of free drugs. (**A**) Evaluation of TMZ, Ver-A, and combined TMZ and Ver-A using U87 and U251 cells. ∆: U251 and **○**: U87. Data represent mean ± SEM, *n* = 3. (**B**) Western blotting analysis of proliferation and apoptosis biomarkers in U87 cells and U251 cells treated with (**B1**) Ver-A or (**B2**) TMZ in combination with Ver-A. M: marker; 1: GBM cells without treatment; 2: GBM cells treated with 2 nM Ver-A or 20 µM TMZ and 2 nM Ver-A.

First, the in vitro anti-GBM cytotoxicity of eight dosages of free drugs, i.e., TMZ (control, standard chemotherapy in clinics) and Ver-A (Figure 4A) were tested with U87 and U251 cells in 96-well plates. The relative cell viabilities were 100%–0.0% for U251 cells and 100%–0.0% for U87 cells post treatment with 0, 10, 25, 50, 75, 100, 150, and 200 µM of free TMZ in the end of cytotoxicity assay. The relative cell viabilities were 100%–0.0% for U251 cells and 100%–3.6% for U87 cells post treatment with 0, 5, 10, 15, 20, 30, 40, and 50 nM of free Ver-A in the end of the assay. The calculated IC_50_ values were 49.2 µM for U251 and 17.5 µM for U87 cells that were treated with TMZ, and 2.1 nM for U251 and U87 cells that were treated with Ver-A. These results indicated that the Ver-A reduced GBM cell growth at low, single-digit nanomolar concentrations in a dose-dependent manner, which was more toxic to GBM cells than TMZ.

Second, to investigate the synergism of standard chemotherapy TMZ and the highly potent Ver-A, we also tested multiple dosages of combined TMZ and Ver-A (Figure 4A). The combined TMZ/Ver-A showed similar cytotoxicity to both U251 and U87 cells as Ver-A only in the tested dosages. The possible reason is that the high potency of Ver-A masked the cytotoxicity of TMZ at low dosages. Since TMZ is the standard chemotherapy for newly diagnosed GBM, we applied TMZ to treat GBM first to mimic clinical application, followed by mAb-EV-Ver-A treatment to evaluate the in vivo anti-GBM efficacy.

Finally, we further investigated the possible anti-tumor mechanism of Ver-A by analyzing the proliferation markers in GBM cell lines (U251 and U87) using Western blotting analysis. The results showed that 2 nM of Ver-A reduced the expression of oncogenic anti-proliferation protein cyclin D1 and increased the expression of cyclin-dependent kinase inhibitors (p21 and p27) at 48 h after treatment (Figure 4B1). These results are consistent with the literature reported anti-cancer mechanism of Ver-A in pancreatic adenocarcinoma and prostate cancer [27,51] and our previous results in neuroendocrine cancer [28]. This study indicated that Ver-A impacts the regulation of proliferation of GBM cells, although the mechanisms need further investigation. As reported in literature [2,3], TMZ is a DNA alkylating agent to induce cell cycle arrest at G2/M and eventually lead to apoptosis, which has different anti-tumor mechanisms from Ver-A. The Western blot analysis of cyclin D1, p21 and p27 in U251 and U87 cells that were treated with 20 µM of TMZ, and 2 nM of Ver-A is presented in Figure 4B2. The analysis of cell cycle phase distribution could further reveal the anti-GBM mechanism of combined TMZ and Ver-A, which will be performed in future. Altogether, the in vitro cytotoxicity study demonstrated that Ver-A is a highly potent payload to GBM.

### 3.5. Tolerated Dosages (TD)

**Figure 5:**
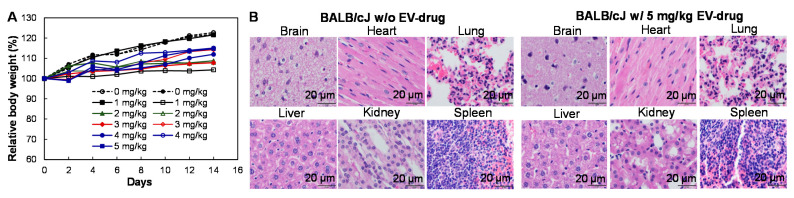
Tolerated dosage (TD) and toxicity analysis of mAb-EV-Ver-A. (**A**) Body weight change of non-GBM carrying BALB/cJ mice after treatment with five dosages of mAb-EV-Ver-A, including 0 (●, **○**), 1 (■, □), 2 (▲, ∆), 3 (♦, ◊), 4 (●, **○**), and 5 (■) mg/kg (*n* = 2). (**B**) H&E staining of main organs, including the brain, heart, lung, liver, kidney, and spleen. Scale bar equals 20 µm.

To investigate the possible toxicity of mAb-EV-Ver-A, 5 different doses of mAb-EV-Ver-A, including 0, 0.5, 1, 2, 3, 4, and 5 mg/kg doses, were injected into BALB/cJ via the tail vein (*n* = 2). During the 14 days post injection, the changes of body weight were in the range of 4.3–22.7% for all the groups (Figure 5A). The H&E staining of the sections of important organs (brain, heart, lung, liver, kidney, and spleen) did not reveal any morphology change or necrosis after mAb-EV-Ver-A treatment, indicating minimal or no toxicity of the targeting delivered Ver-A (Figure 5B). Two dosages of mAb-EV-Ver-A (1 and 3 mg/kg), which did not show toxicity, were used in the following in vivo anti-tumor efficacy study. The low n value was used in this study and one mouse was lost by accident from a dosage of 5 mg/kg, so further toxicology studies are needed to make a statistically significant conclusion in the future.

### 3.6. In Vivo Anti-GBM Efficacy in Intracranial Xenograft Model

**Figure 6:**
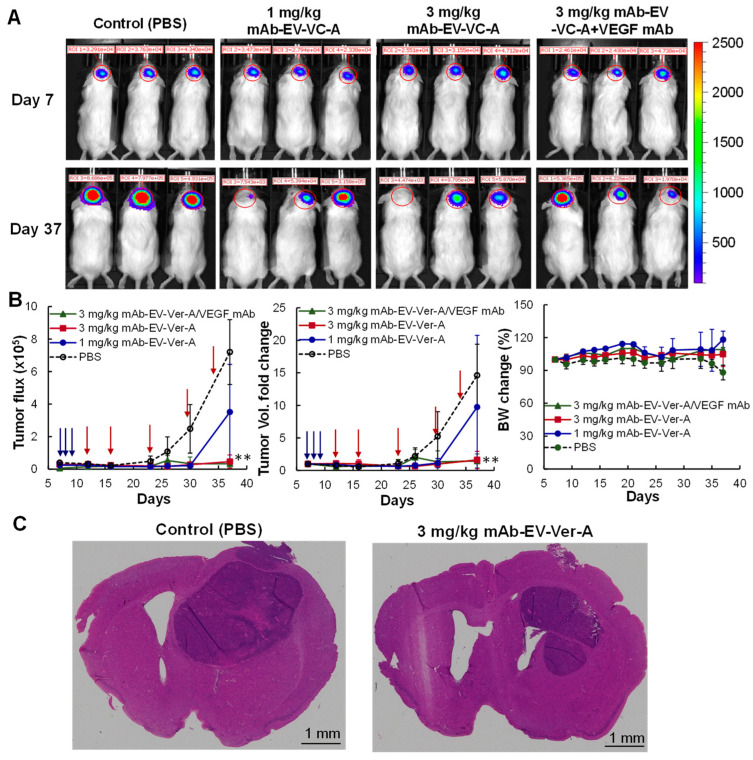
In vivo evaluation of the anti-GBM efficacy of mAb-EV-Ver-A. All mice were treated with 1 mg/kg TMZ daily on days 7–12. (**A**) Representative IVIS images of GBM intracranial xenograft mice treated with EGFR mAb-EV-Ver-A on days 12, 16, 23, 30, and 34. (**B**) Tumor flux, volume fold change, and body weight profiles. Tumor growth was monitored by measuring the FLuc bioluminescence using IVIS, and body weight was measured every 3–4 days. The blue arrows indicate the I.P. administration of TMZ. The red arrows indicate the I.V. administration of mAb-EV-Ver-A. ○: PBS (control); ●: 1.0 mg/kg mAb-EV-Ver-A; ■: 3.0 mg/kg mAb-EV-Ver-A; and ▲: 3.0 mg/kg mAb-EV-Ver-A in combination with VEGF mAb injection on Q3/7Dx7. ** *p* < 0.005 vs. control using ANOVA followed by Dunnett’s *t*-test. Data represent mean ± SEM, *n* = 5. (**C**) Representative H&E staining of brain tissue section. Scale bar: 1 mm.

To evaluate the in vivo treatment efficacy of the targeted delivery of Ver-A by mAb-EV, we generated U87-FLuc intracranial xenograft models using 6-week NSG mice. When tumor volume reached bioluminescent intensity of over 20,000 on day 7 as detected by IVIS imaging, the xenografted mice were treated with 1 mg/kg of TMZ daily on days 7–9 to mimic the clinical treatment. Then, the mice were further treated with PBS (negative control), 1.0 mg/kg mAb-EV-Ver-A, 3.0 mg/kg mAb-EV-Ver-A, and 3.0 mg/kg mAb-EV-Ver-A in combination with VEGF mAb (positive control) via i.v. injection on days 9, 12, 16, 23, 30, and 34 post cell implantation. The tumor volume was monitored by measuring fluorescent flux with IVIS. The representative IVIS images captured pre- and post-mAb-EV-Ver-A injection are presented in Figure 6A. Both absolute tumor flux and relative tumor volume fold change are described in Figure 6B. Specifically, the GBM tumor growth was significantly inhibited in the 1 mg/kg and 3 mg/kg of mAb-EV-Ver-A treatment groups as compared to the control group (*p* ≤ 0.005) during the treatment period. The combination with anti-VEGF mAb did not improve the treatment efficacy. In addition, there was no obvious body weight difference among all the four groups (Figure 6B), indicating minimal side toxicity. The treatment was terminated when the control group showed obvious slow locomotion and body weight loss (>20%). The harvested brain tumor tissues were sectioned to perform H&E staining to further confirm the anti-GBM efficacy. The H&E images demonstrated an obvious reduction of the tumor burden with the treatment of mAb-EV-Ver-A (Figure 6C). These findings support the hypothesis that mAb-EV-Ver-A can effectively target the GBM xenograft and successfully deliver drugs for GBM treatment.

## 4. Discussion

The combination of surgery, radiotherapy, and chemotherapy is still the standard care to treat GBM in clinics. Targeted therapies driven by the tumor or immune biomarkers have been demonstrated promise in preclinical GBM models. However, as reviewed in literature [5,52], an extensive number of targeted therapies have failed in clinical trials because of the insufficient inhibition of signaling pathway, poor drug delivery efficiency, or tumor heterogeneity. Our research identified and evaluated a new drug candidate (Ver-A) as an adjuvant therapy post primary treatment and established a targeted delivery vehicle (mAb-EV) that could penetrate the BBB, target GBM cells, and effectively inhibit tumor growth as well as minimize systemic toxicities. To better capture the GBM tumor microenvironment, we are developing the patient-derived xenograft model, which could be used to further evaluate the new therapy in future studies.

This study demonstrated that Ver-A can kill ~100% cells of multiple GBM lines and inhibit tumor growth and reduce tumor volume in a GBM intracranial xenograft model post TMZ treatment (mimicking clinic treatment). Ver-A has multiple anti-tumor mechanisms, such as inhibition of proliferation, block of cell cycle in the S phase, depolarization of mitochondria, and induction of apoptosis, so it could be an ideal drug candidate or an adjuvant of standard chemotherapy of DNA alkylating TMZ in GBM treatment. In addition to the high potency, these integrated anti-tumor mechanisms of Ver-A could reduce the possibility of drug resistance development during long-term treatment. In the future, we will further investigate the anti-GBM mechanisms of Ver-A. Moreover, Ver-A is a natural compound and has low systemic toxicity.

Although multiple drug delivery vehicles (liposome, polymeric nanoparticle, solid lipid, and polymeric micelle) have been developed in cancer therapy, it is very challengeable to use these synthesized nanoparticles in GBM treatment due to the issues of stability and/or the BBB [29]. As a natural vesicle, EV has multiple advantages over the synthesized particles, including low immune toxicity, high stability, and efficient penetration of the BBB [36,37]. This study used our established biomanufacturing platform [28,38] to generate EV from human cell line, surface-tagged mAb, and the packed drug Ver-A, which demonstrated the capabilities of high GBM targeting and effective drug delivery. Importantly, different mAbs (single or multiple) can be easily conjugated to the surface of EV to target the well-known or newly identified receptors in GBM or angiogenesis to cross the BBB.

The literature [45,53,54,55] and our immunohistochemistry staining of GBM patient tissue microarray show that EGFR or EGFRvIII is overexpressed in more than 40% GBM patients. The chimeric anti-EGFR mAb, Cetuximab, has been approved by the U.S FDA and used to treat head and neck cancer [56,57] and colorectal cancer [58,59]. The therapies to target EGFR signaling pathways have limited clinical benefits in GBM treatment [60,61,62], but EGFR has been demonstrated as a good surface target, and the anti-EGFR mAb or peptide can direct the targeted delivery of therapies to GBM [45,63,64,65,66]. The anti-EGFR mAb-EV-Ver-A constructed in this study showed high anti-GBM efficacy, indicating EGFR as a good surface receptor to target GBM. To treat the EGFR-GBM, we can tag the mAbs to target other surface receptors overexpressed in GBM, such as PDGFR, TGF-βR, CXCR4, uPAR, and others as reported in the literature [43,44].

The anti-VEGF mAb, Bevacizumab, remains the only U.S FDA-approved molecular therapy to treat recurrent GBM. The clinical data showed that Bevacizumab can improve the progression-free survival but has no benefit to improve overall survival [67]. In this study, we tested the combined mAb-EV-Ver-A and anti-VEGF mAb but observed no benefit of VEGF mAb in GBM treatment.

In addition to chemotherapy, immunotherapies, such as CAR-T [68,69,70,71] and immune checkpoint inhibitors of cytotoxic T lymphocyte-associated protein 4 (CTLA-4), programmed cell death protein 1 (PD-1), and programmed cell death protein 1 ligand (PD-L1) [72,73,74], have been recently investigated for GBM treatment. Moreover, induced pluripotent stem cell-based regenerative medicine [75] and gene therapies (adenovirus, herpes simplex virus-1, retrovirus, shRNA, siRNA, non-viral vectors) have been developed and evaluated [76,77,78,79]. We will evaluate the combination of our mAb-EV-Ver-A and immunotherapy or gene therapy for GBM treatment in the future.

## 5. Conclusions

The targeted mAb-EV has great potential to deliver chemotherapy to treat the highly aggressive glioblastoma because of the capability to cross the blood–brain barrier, specificity of tumor targeting, and advantage to deliver combined therapies such as chemotherapy, gene therapy, and others. The natural compound, verrucarin A, has a high potency and low systemic toxicity in glioblastoma treatment, which could provide a new therapy for patients. Despite these promising results, the developed mAb-EV-Ver-A needs further pre-clinical evaluations such as pharmacokinetics, pharmacodynamics, and immune modulatory responses in the future.

## Data Availability

The data presented in this study are contained within the article.
