# Correction: Chen et al. Targeted Extracellular Vesicles Delivered Verrucarin A to Treat Glioblastoma. *Biomedicines* 2022, *10*, 130

**DOI:** 10.3390/biomedicines12010086

**Published:** 2023-12-29

**Authors:** Kai Chen, Yingnan Si, Jia-Shiung Guan, Zhuoxin Zhou, Seulhee Kim, Taehyun Kim, Liang Shan, Christopher D. Willey, Lufang Zhou, Xiaoguang Liu

**Affiliations:** 1 Department of Biomedical Engineering, University of Alabama at Birmingham (UAB), 1825 University Blvd, Birmingham, AL 35294, USA; kaisdzb@uab.edu (K.C.); yingnan@uab.edu (Y.S.); zhouzhx@uab.edu (Z.Z.); 2 Department of Medicine, University of Alabama at Birmingham (UAB), 703 19th Street South, Birmingham, AL 35294, USA; guan0926@uab.edu (J.-S.G.); seulheekim@uabmc.edu (S.K.); kimth@uab.edu (T.K.); 3 School of Nursing, University of Alabama at Birmingham (UAB), 1701 University Blvd, Birmingham, AL 35294, USA; lshan@uab.edu; 4 Department of Radiation Oncology, University of Alabama at Birmingham (UAB), 1700 6th Avenue South, Birmingham, AL 35294, USA; cwilley@uabmc.edu

## Error in Figure

In the original publication [1], there was a mistake in Figure 6. In vivo evaluation of the anti-GBM efficacy of mAb-EV-Ver-A as published. The incorrect baseline IVIS image was used. The corrected Figure 6. In vivo evaluation of the anti-GBM efficacy of mAb-EV-Ver-A appears below. The original publication has been updated to reflect the author’s current institutional email address. The authors state that the scientific conclusions are unaffected. This correction was approved by the Academic Editor. The original publication has also been updated.

## Figures and Tables

**Figure 6:**
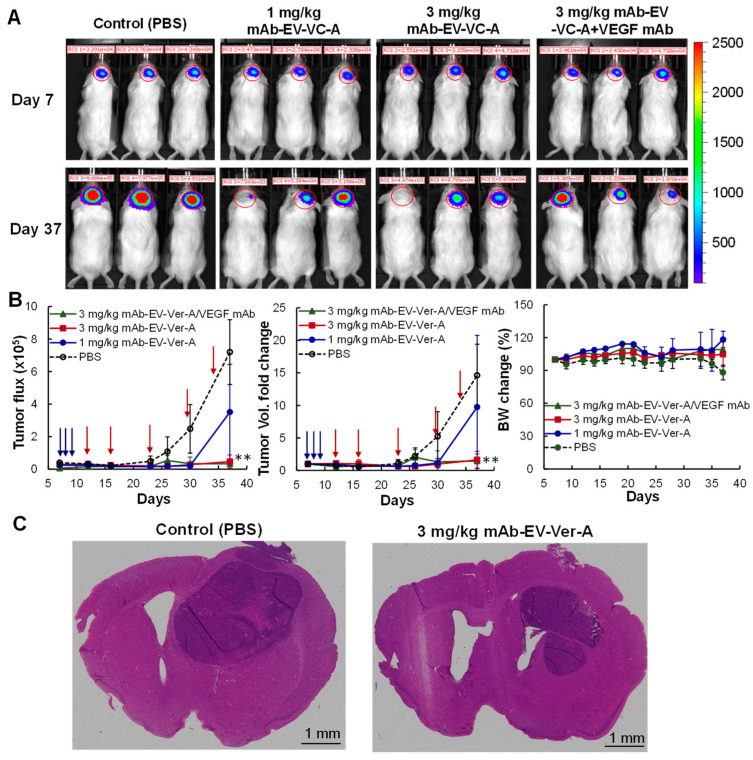
In vivo evaluation of the anti-GBM efficacy of mAb-EV-Ver-A. All mice were treated with 1 mg/kg TMZ daily on days 7–12. (**A**) Representative IVIS images of GBM intracranial xenograft mice treated with EGFR mAb-EV-Ver-A on days 12, 16, 23, 30, and 34. (**B**) Tumor flux, volume fold change, and body weight profiles. Tumor growth was monitored by measuring the FLuc bioluminescence using IVIS, and body weight was measured every 3–4 days. The blue arrows indicate the I.P. administration of TMZ. The red arrows indicate the I.V. administration of mAb-EV-Ver-A. ○: PBS (control); ●: 1.0 mg/kg mAb-EV-Ver-A; ■: 3.0 mg/kg mAb-EV-Ver-A; and ▲: 3.0 mg/kg mAb-EV-Ver-A in combination with VEGF mAb injection on Q3/7Dx7. ** *p* < 0.005 vs. control using ANOVA followed by Dunnett’s *t*-test. Data represent mean ± SEM, *n* = 5. (**C**) Representative H&E staining of brain tissue section. Scale bar: 1 mm.
